# Influence of Long-Term CaO Storage Conditions on the Calcium Looping Thermochemical Reactivity

**DOI:** 10.1021/acs.energyfuels.3c02652

**Published:** 2023-10-20

**Authors:** Nabil Amghar, Antonio Perejón, Carlos Ortiz, Luis A. Pérez Maqueda, Pedro E. Sánchez-Jiménez

**Affiliations:** † Instituto de Ciencia de Materiales de Sevilla (C.S.I.C.-Universidad de Sevilla). C. Américo Vespucio 49, Sevilla 41092, Spain; ‡ Departamento de Química Inorgánica, Facultad de Química, Universidad de Sevilla, Sevilla 41012, Spain; § Materials and Sustainability Group, Department of Engineering, Universidad Loyola Andalucía, Avda. de las Universidades s/n, Dos Hermanas 41704, Seville, Spain

## Abstract

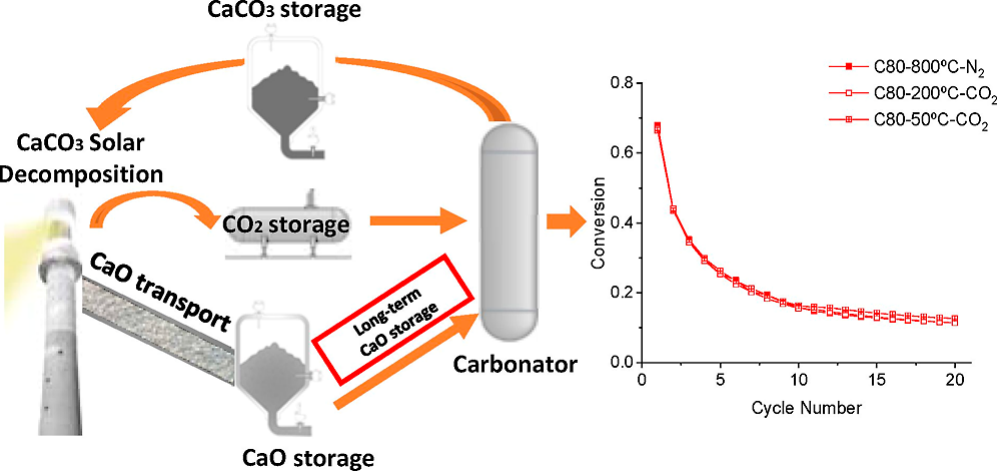

Long-term storage capability is often claimed as one of the distinct advantages of the calcium looping process as a potential thermochemical energy storage system for integration into solar power plants. However, the influence of storage conditions on the looping performance has seldom been evaluated experimentally. The storage conditions must be carefully considered as any potential carbonation at the CaO storage tank would reduce the energy released during the subsequent carbonation, thereby penalizing the round-trip efficiency. From lab-scale to conceptual process engineering, this work considers the effects of storing solids at low temperatures (50–200 °C) in a CO_2_ atmosphere or at high temperatures (800 °C) in N_2_. Experimental results show that carbonation at temperatures below 200 °C is limited; thus, the solids could be stored during long times even in CO_2_. It is also demonstrated at the lab scale that the multicycle performance is not substantially altered by storing the solids at low temperatures (under CO_2_) or high temperatures (N_2_ atmosphere). From an overall process perspective, keeping solids at high temperatures leads to easier heat integration, a better plant efficiency (+2–4%), and a significantly higher energy density (+40–62%) than considering low-temperature storage. The smooth difference in the overall plant efficiency with the temperature suggests a proper long-term energy storage performance if adequate energy integration is carried out.

## Introduction

The calcium looping (CaL) process has been intensely investigated in the last years as a promising system for thermochemical energy storage (TCES).^1−4^ It is based on the reversible reaction between CO_2_ and CaO to form CaCO_3_ (eq 1)
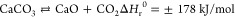
1

The use of CaL as a mid to high-temperature carbon capture and storage technology is currently at the technology readiness level (TRL) 7.^5,6^ As a TCES system, CaL displays several advantages, such as high energy density, nontoxicity, and the wide availability and affordability of the potential raw materials, which include Ca-containing minerals, rocks, and even industrial wastes.^2,7−10^

TCES based on CaL has been typically considered to be integrated into concentrating solar power (CSP) due to the compatible charge and discharge temperatures.^11,12^ Solar radiation drives the endothermic decomposition of CaCO_3_ into CaO and CO_2_ in a solar reactor.^3,13^ The reaction products are separately conducted to storage reservoirs and then brought back together in the carbonator reactor on demand for energy production. The heat released in the reverse exothermic reaction is exploited in a power cycle (i.e., CO_2_-closed Brayton cycle) to produce electricity.^14,15^ The equilibrium temperature in a CO_2_ atmosphere at 1 bar is ∼895 °C.^16^ Consequently, for achieving fast calcination in CO_2_, the reaction temperature has to be maintained above ∼950 °C.^17,18^ The carbonation reaction is normally proposed at ∼800–850 °C to ensure a rapid reaction and high thermoelectric efficiency.^14,19,20^ High temperature and CO_2_-rich environments substantially promote the grain growth and sintering of CaO particles, which, consequently, speed up the deactivation of CaO.^18,21−23^ Thus, the calcination stage is frequently carried out under an inert gas or at reduced pressure, which allows the reduction of the minimum required temperature to 750 °C.^24−26^

A literature review of recent studies on the CaL system shows a profound interest in sorbent properties, reactor design and operation, process integration, and economic analyses.^27−30^ Lately, there has been progress in the design of more efficient and potentially cost-effective plant schemes. Multiple options have been contemplated, such as fuel consumption and equipment size reduction,^31,32^ improvement in heat transfer from hot calcination products to colder carbonation sorbents,^33,34^ reactivation of the sorbent,^35^ and integration with solar technologies.^13,36−38^

One of the key advantages of CaL as a TCES system is the possibility of long-term energy storage. Current state-of-the-art thermal storage (TES) technologies are mainly based on molten salts. In this case, the need to trace the system to maintain temperatures over 200 °C to avoid the solidification of the molten salts entails a substantial increase in costs.^27,39^ Many process schemes in the literature consider either high temperature or low temperature solids storage for CaL integration.^27,40−42^ However, none of the studies published in the literature have yet considered the influence of the storage conditions on the CaO reactivity, and only a few studies have directly compared the effect of storing the solids at high or low temperatures.^43−46^ Understanding the storage stage influence is crucial to optimize the whole process. An excessively high storage temperature of the reactants might deteriorate the CaO reactivity due to sintering.^18,47^ The atmosphere is also relevant because of the high reactivity of CaO with H_2_O and CO_2_. Thus, even small contents of moisture and CO_2_ might partially convert CaO into Ca(OH)_2_ or CaCO_3_ during the storage period, thereby incurring undesired energy release during the storage step.^48^

The present work explores the impact of the storage conditions of the reactants on the multicycle activity of CaO for TCES. While previous research has evaluated the influence of using different storage temperatures for the CaO silos, there are no studies considering the influence on CaO multicycle performance. Thus, the performance of limestone has been evaluated when a storage step is inserted between the calcination and carbonation cycles. Three different storage temperatures have been explored; 50 and 200 °C under CO_2_ and 800 °C under N_2_. This work also contemplates the influence of critical parameters such as temperature, atmosphere, particle size, and time. Finally, the impact of the storage temperature over the round trip efficiency is also assessed from a process engineering perspective.

## Materials and Methods

### Materials

Limestone was provided by KSL Staubtechnik GmbH from the standard Eskal series. According to the supplier, the limestone has 99.1% content in CaCO_3_ but also contains as impurities 0.45% MgO, 0.25% SiO_2_, 0.1% Al_2_O_3_, and 0.04% Fe_2_O_3_. Samples with two well-defined particle sizes were used: 80 and 150 μm. These particle sizes were selected considering that particles below 50 μm cannot be fluidized in the proposed practical application due to their cohesiveness.^49−51^ The samples are referred to hereafter as C80 and C150, respectively.

**Figure 1:**
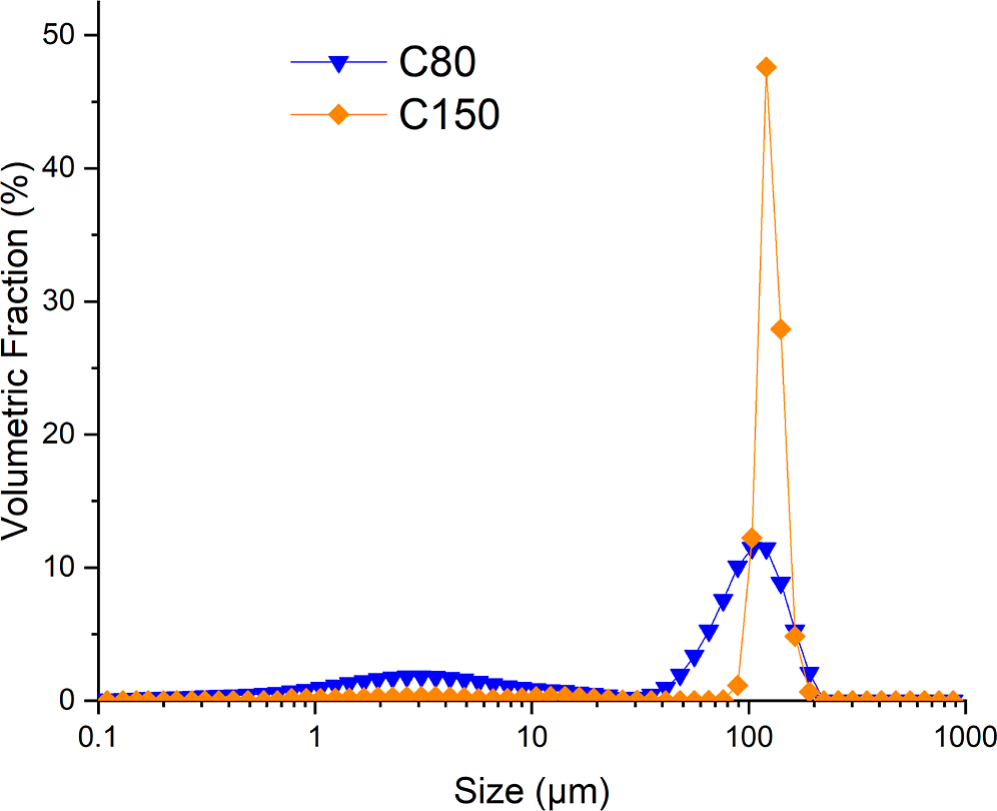
PSD data measured for C80 and C150.

Particle size distribution (PSD) data are listed in Table 1, while the frequency distributions of the particle sizes for the samples are plotted in Figure 1. As can be seen, the samples present a narrow PSD.

**Table 1:** PSD Parameters of the Two Limestone Samples^a^

PSD data (μm)			
sample	Dv(10)	Dv(50)	Dv(90)
C80	1.60	74.91	133.47
C150	113.11	133.60	157.24

a Dv(10), Dv(50), and Dv(90) indicate the percentiles, meaning that 10, 50, and 90% of the sample is smaller than the given size, respectively.

## Experimental Methods

The multicycle activity was studied in a thermogravimetric analyzer Q5000 developed by TA Instruments, equipped with a high sensitivity balance (<0.1 μm). This instrument allows for high heating and cooling rates (∼300 °C/min) from room temperature to 1000 °C, using infrared halogen lamps that heat the silicon carbide furnace where the sample is placed. These high heating rates are necessary to simulate realistic Ca-Looping conditions in which the material undergoes calcination and carbonation under different atmospheres and temperatures.

**Figure 2:**
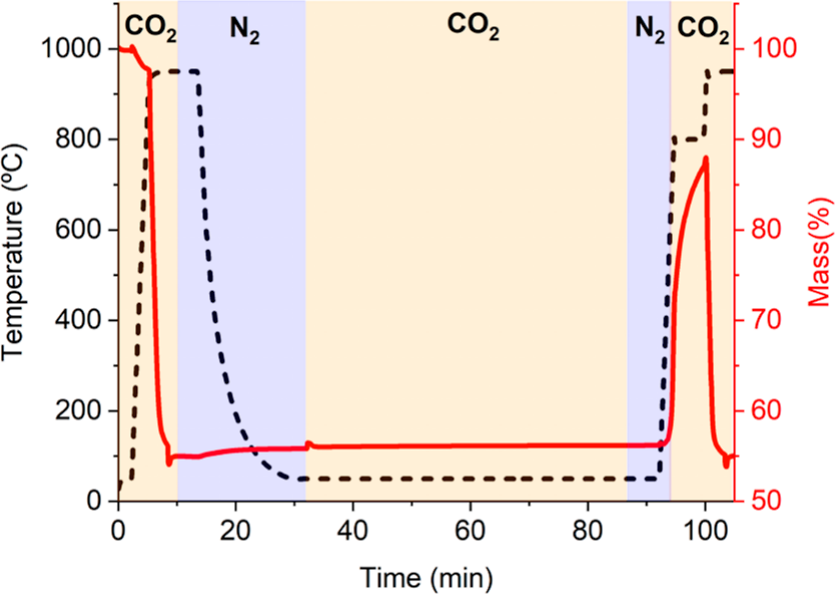
Schematic diagram of the experimental procedure for evaluating storage tests. Blue and yellow indicate which experiment segments were carried out in N_2_ or CO_2_ atmospheres, respectively.

Two experimental schemes were used in this work. The first one comprises an initial heating step to a calcination temperature of 950 °C in a CO_2_ atmosphere. The calcination temperature is maintained for 3 min. Then, the atmosphere is switched to N_2,_ and purged for 5 min to ensure the complete removal of CO_2_ from the system. This is done to avoid carbonation while cooling to the storage step in order to correctly assess the results and discriminate the influence of CO_2_ in the storage step. The system is cooled to the desired storage temperature (200 and 50 °C). Once the storage temperature is achieved, the atmosphere is again switched to CO_2_ and maintained for the planned storage time. To evaluate the influence of storage on subsequent carbonation stages, the sample is heated in nitrogen to a carbonation temperature of 800 °C at 300 °C/min, when the atmosphere is changed to CO_2_. For example, Figure 2 shows a scheme of the test involving storage in CO_2_ at 50 °C.

The second scheme represents a CSP CaL process scheme in which solids are stored at high temperatures to simplify the reactors’ thermal integration.^15^ The storage step is introduced at high temperatures in a N_2_ atmosphere between the calcination and carbonation stages. The calcination is performed at 950 °C in CO_2_, and then, the atmosphere is switched to N_2_. The system is then cooled to a storage temperature (800 °C), which lasts 1 h in this atmosphere. Then, to proceed with the multicycle tests, the atmosphere is changed to CO_2_, and the carbonation is triggered at 850 °C during 5 min. Table 2 summarizes the experimental conditions for the evaluation of the multicycle performance used in this work.

**Table 2:** Experimental Conditions for the Different Calcination/Carbonation Tests Carried Out in This Work

	calcination			carbonation			storage		
test	*T*, °C	*t*, min	gas	*T*, °C	*t*, min	gGas	*T*, °C	*t*, min	gas
C80-50 °C-CO_2_	950	5	CO_2_	800	5	CO_2_	50	60	CO_2_
C80-200 °C-CO_2_	950	5	CO_2_	800	5	CO_2_	200	60	CO_2_
C80-800 °C-CO_2_	950	5	CO_2_	800	5	CO_2_	800	60	N_2_
C150-50 °C-CO_2_	950	5	CO_2_	800	5	CO_2_	50	60	CO_2_
C150-200 °C-CO_2_	950	5	CO_2_	800	5	CO_2_	200	60	CO_2_
C150-800 °C-CO_2_	950	5	CO_2_	800	5	CO_2_	800	60	N_2_

The PSD was measured by laser diffraction using a Mastersizer 2000 from Malvern. The samples were sonicated for 30 min and dispersed in distilled water to avoid aggregation.

### CaO Conversion and Residual CaO Conversion

The conversion of CaO to CaCO_3_ is the main parameter used in this work to evaluate the multicycle performance of the samples. The total CaO conversion (*X*_T,N_) in each cycle is defined as the sum of the CaO conversion during storage (*X*_sto_) (which is undesirable since the heat is not being released to the power cycle) and the conversion in the subsequent carbonation stage (*X*_CaO,N_)
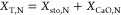
2

Moreover, *X*_T,N_ may be expressed as
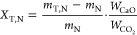
3where *m*_T,N_ is the total sample mass after the storage step and the subsequent carbonation stage and m_N_ is the sample mass after calcination (before the storage starts). *W*_CaO_ and *W*_CO_2__ are the molar masses of CaO and CO_2_, respectively.

The undesired conversion of CaO that would take place during the storage step is
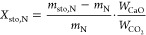
4where *m*_sto_ is the sample mass after the storage step at the *Nth*-cycle. *X*_sto_ considers the nondesired reaction of CaO with CO_2_ during storage. The heat released during this step would be wasted.

From the above equations, the CaO conversion in the carbonation stage of the cycles can be obtained once the values of *X*_T,N_ and *X*_sto,N_ are calculated
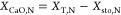
5

### Energy Storage Density of the Calcined Material

The energy storage density (in GJ/m^3^) of the calcined materials can be quantified from the energy density per mass
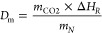
6

Here, *m*_CO_2__ is the CO_2_ uptake during carbonation, computed from the CaO conversion data, Δ*H*_R_ is the enthalpy of the reaction per kg of CO_2_ (4045.5 kJ/kg CO_2_), and *m*_N_ is the sample mass after calcination. The energy storage density is then calculated from eq 8
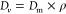
7where ρ is the density of the calcined material, assuming a porosity of 50% (for CaO, it results in a density of 1670 kg/m^3^). Given this value, the maximum theoretical volumetric energy density for CaO would be 3.7 GJ/m^3^.

## Results and Discussion

### Effect of Storage in CO_2_ at Different Temperatures

Optimum storage conditions for CaO are essential to ensure a proper plant’s overall performance. The storage temperature imposes rules for thermal energy integration of the reactors. Besides, the material’s reactivity during the storage step should also be considered an essential parameter that could reduce the available energy released into the power cycle.

**Figure 3:**
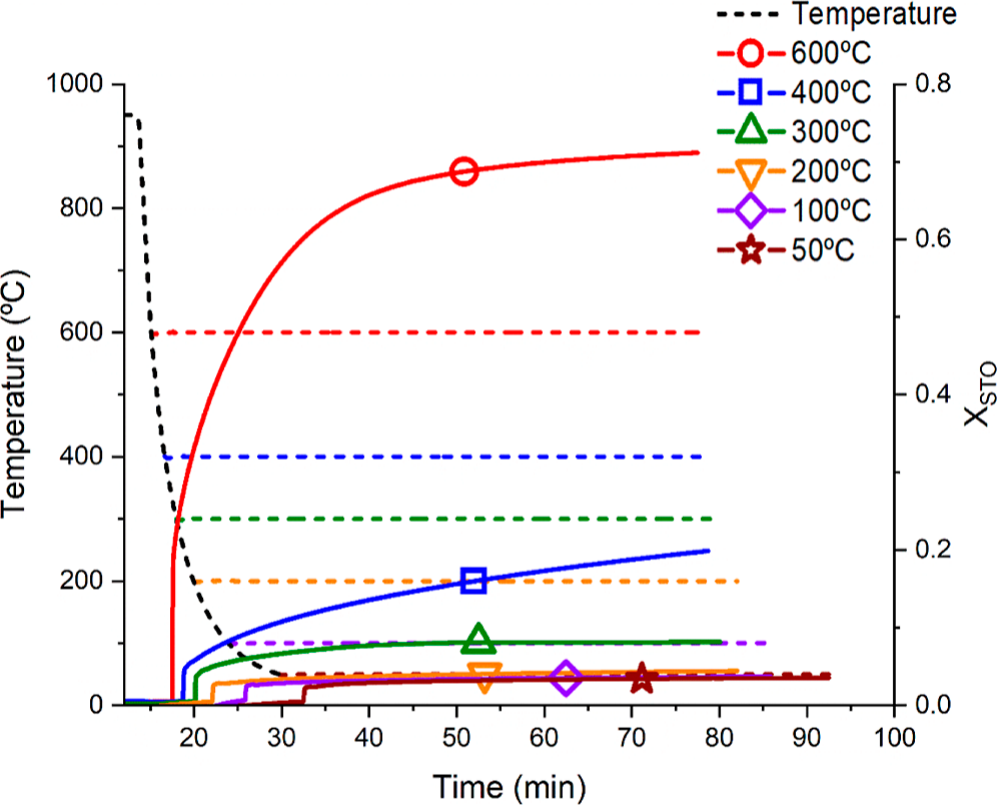
Time evolution of the storage conversion for calcined C80 samples, maintained in a CO_2_ atmosphere at different temperatures.

Figure 3 shows the behavior during storage of CaO derived from the decomposition of C80 limestone particles at different temperatures under a CO_2_ atmosphere. Thus, in these experiments, a freshly calcined sample is rapidly cooled down to a set storage temperature. Only when the desired temperature is reached, CO_2_ is injected into the system (as described in Figure 2). Almost immediately after CO_2_ injection, the sample mass increases due to the carbonation. Two carbonation stages are evident; a very fast reaction-controlled stage followed by a slower, diffusion-controlled carbonation stage.^21,52,53^ The higher the temperature, the more significant the fraction of CaO that converts into CaCO_3_ during the fast carbonation stage. Likewise, diffusion-controlled carbonation is equally promoted at higher temperature. It is evident from Figure 3 that storing the solids under pure CO_2_ at 600 and 400 °C is undoubtedly detrimental as the carbonation reaction is kinetically very favored at these temperatures. A storage temperature of 600 °C approaches the conditions used in CO_2_ capture applications, where carbonation occurs at 650 °C under a less rich CO_2_ atmosphere (∼10–15% vol.).^54,55^ On the other hand, it appears that at temperatures below 200 °C, the carbonation results in essentially similar values of CaO conversion (*X*_sto_ = 0.04). Thus, cooling the CaO all the way down to room temperature might not be necessary to preserve the reactivity of the material for subsequent carbonation cycles.

Consequently, storage at temperatures higher than 200 °C must be avoided if the material is stored under a pure CO_2_ atmosphere. The storage temperature of 200 °C can be used as a proper comparison with the current molten salt storage temperature,^56^ while the storage temperature at 50 °C is a reference for room temperature storage. Remarkably, in the case of CaO particles, there are no significant issues if particles are cooled from 200 °C, so there is no critical process limitation as in the case of molten salts.

### Effect of Storage Time in CO_2_

**Figure 4:**
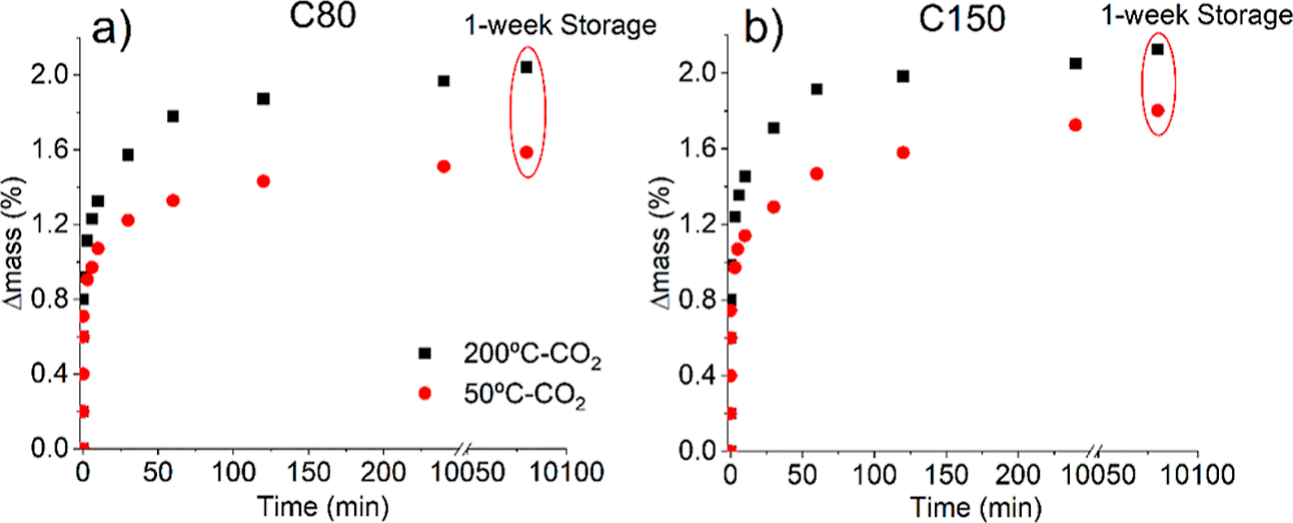
Mass percentage gains as a function of time for samples tested at 50 and 200 °C in CO_2_. (a) C80 and (b) C150. Legend is shared for both graphics and represents the temperature of the storage step. The red ellipse highlights the value for 1 week storage.

The storage of the reaction products is expected to last for at least some hours (if not days, as potentially occurred in TCES systems). Consequently, the time that CaO can be stored without activity loss in subsequent cycles is relevant to the overall process efficiency. Figure 4 shows the time evolution of the mass percentage gained during the storage step in CO_2_ for C80 and C150 limestone particles. For this study, an experiment similar to that presented in Figure 2 was performed but with extended storage times. Two different storage temperatures were compared: 50 and 200 °C.

As seen in Figure 4, the main part of the overall conversion occurs within the first 30–50 min. Moreover, about half of the total mass gain occurs during the first 6 s after CO_2_ is injected, regardless of particle size. Noticeably, the observed behavior is similar to the carbonation profiles at higher temperatures: a fast controlled reaction phase lasts only a few seconds, followed by a slower diffusion-controlled reaction phase.^21,52,53^ It can be inferred from the experiments in Figure 4 that long-term storage is feasible as the loss of active material is very small due to the limited fast controlled reaction phase at 50 and 200 °C and the very slow diffusion-controlled carbonation kinetics. Note that after 1 week, the mass percentage gained is still lower than 2.2%, indicating that long-term storage at 50 and 200 °C is feasible even in a CO_2_ atmosphere. Considering these results, a storage time of 60 min was used in the subsequent experiments since higher solids storage times would not substantially alter the obtained results.

### Effect of the Storage Step on the Multicycle Performance

**Figure 5:**
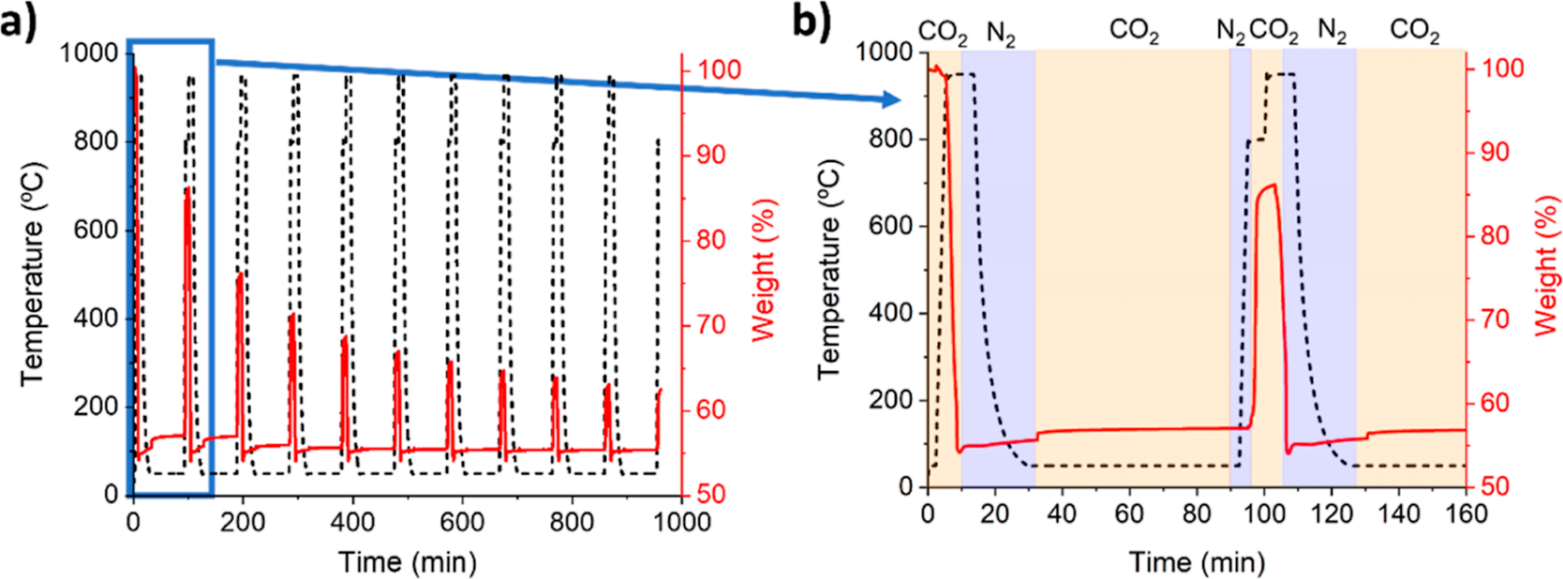
(a) Time evolution of temperature and sample mass (C80 sample) recorded in the thermogravimetric analysis during multicycle calcination/carbonation tests using a 60 min storage step at 50 °C. (b) Close-up view of the first cycle. Calcination and carbonation were carried out in a CO_2_ atmosphere for 5 min at 950 and 800 °C, respectively. Blue and yellow highlight the segments under N_2_ or CO_2_ atmospheres, respectively.

Figure 5a displays the time evolution of the mass along multiple calcination–carbonation cycles recorded for C80 particles. The multicycle scheme includes a 60 min storage step at 50 °C after each calcination stage. Figure 5b corresponds to a close view of the first cycle; it shows that the calcination temperature of 950 °C ensures a rapid conversion of CaCO_3_ into CaO. A progressive deactivation of CaO toward carbonation as the number of cycles increases is evident in Figure 5a due to sintering, which is further promoted in the CO_2_-rich atmosphere because of the higher temperature required for calcination.^18,23^

**Figure 6:**
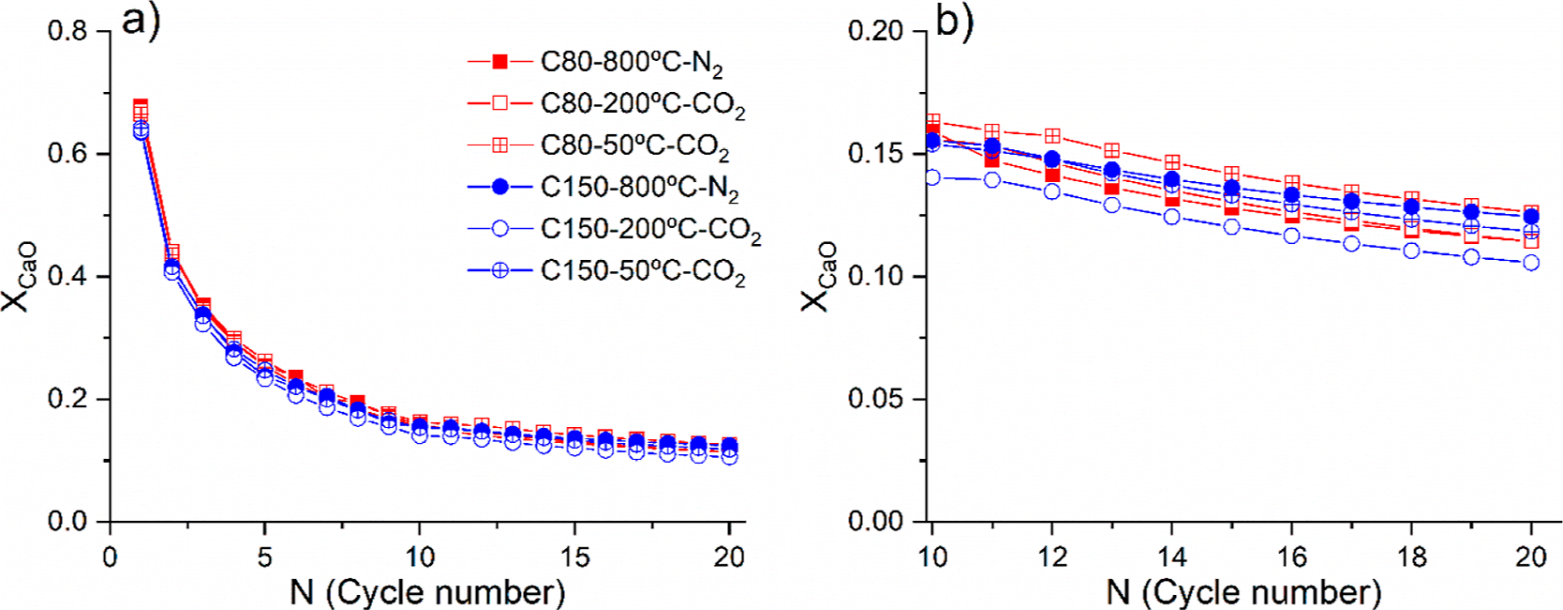
(a) Multicycle evolution of the CaO conversion, calculated from eqs 4 and 5, for C80 and C150. (b) Close-up of the last 10 cycles for C80 and C150. Legend is shared for both graphs. Unfilled symbols represent samples submitted to the 200 °C storage step, and cross-filled symbols represent samples submitted to the 50 °C storage step. Solid symbols represent CaO conversion when a storage step at a high temperature (800 °C) in N_2_ is considered (5 min calcination and carbonation at 950 and 800 °C, respectively).

Figure 6 compares the multicycle performance in terms of CaO conversion (*X*_CaO_) for C80 and C150. CaO conversions are calculated from the thermogravimetry experiments according to eq 5. Three different storage conditions were explored: (i) 50 °C in CO_2_, (ii) 200 °C in CO_2_, and (iii) 800 °C in N_2_. It can be readily concluded from the plots that the overall behavior of the materials does not depend on particle size in the range 80–150 μm. In all three operating conditions studied, the CaO conversions dropped from about 0.65–0.70 in the first cycle down to about 0.17 after 20 calcination and carbonation cycles. Table 3 lists the CaO conversion attained at the 1st and 20th cycles for C80 and C150 under the different experimental conditions. The conversion values obtained agree with previous reports for similar particle sizes.^57^ These results demonstrate that the samples present a similar behavior regardless of the storage conditions implemented.

**Table 3:** Values of CaO Conversion for the 1st (*X*_CaO,1_) and 20th (*X*_CaO,20_) Cycles

sample	storage 200 °C (CO_2_)		storage 50 °C (CO_2_)		storage 800 °C(N_2_)	
	*X*_CaO,1_	*X*_CaO,20_	*X*_CaO,1_	*X*_CaO,20_	*X*_CaO,1_	*X*_CaO,20_
C80	0.670	0.114	0.665	0.126	0.678	0.114
C150	0.637	0.106	0.642	0.118	0.635	0.124

**Figure 7:**
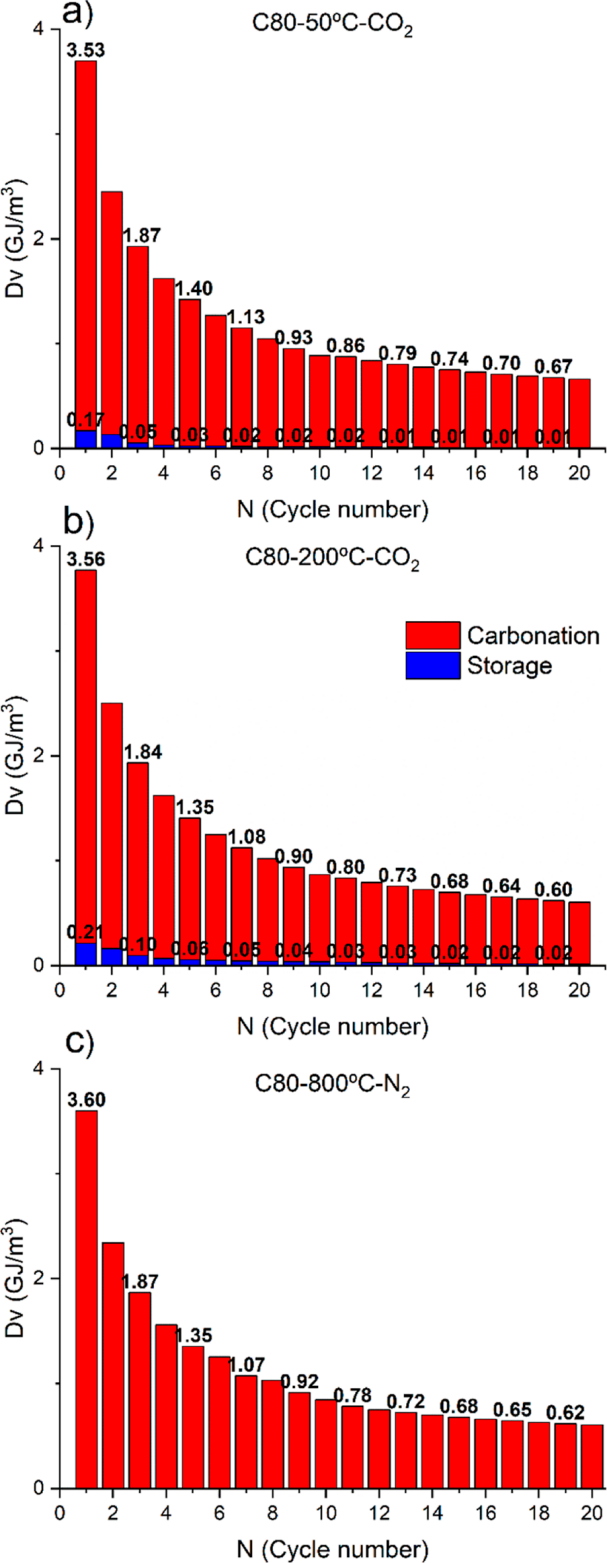
Volumetric energy density values as a function of the cycle number for C80 particles tested by including in the multicycle experiment a storage step at (a) 50 and (b) 200 °C in CO_2_ and (c) 800 °C in N_2_. Values were calculated using eq 7.

Figure 7 compares the results obtained in terms of volumetric energy density, calculated as described in eqs 6 and 7. Red bars correspond to the values calculated using the CaO conversion during the carbonation stage, *X*_CaO_, that is, the energy that can be recovered. On the other hand, blue bars account for the fraction of the energy density wasted during the storage step. Both storage conditions in CO_2_ (200 and 50 °C) yield good results in terms of the capability to preserve the CaO reactivity. As the reactivity of the material decreases with the subsequent cycles, so does the fraction of material that reacts during the storage step. Thus, while non-negligible at a low cycle number, after 5 cycles, the CaO becomes unreactive at low temperatures, and the energy lost during storage becomes negligible. Obviously, in the experiments testing storage in N_2_, there is no energy wasted during storage, as this is implemented in an inert atmosphere.

Table 4 includes the accumulated energy density, calculated as the sum of the energy density (*D*_v_) of each of the 20 calcination/carbonation cycles performed. The energy density values obtained are roughly similar for C80 particles. On the other hand, C150 particles exhibit worse long-term performance when stored in CO_2_ at 200 °C.

**Table 4:** Accumulated Volumetric Energy Density of Limestone Samples with a Storage Step at 200 and 50 °C in CO_2_ and 800 °C in an Inert Atmosphere (N_2_)

sample	*D*_v_ (GJ/m^3^)					
	storage 200 °C (CO_2_)		storage 50 °C (CO_2_)		storage 800 °C(N_2_)	
	carbonation	storage	carbonation	storage	carbonation	storage
C80	22.28	1.28	23.36	0.62	22.65	
C150	20.47	1.34	22.01	0.67	22.20	

### Considerations on the Industrial-Scale TCES Process Integration

This section broadens the focus of the study to assess how a given storage condition can affect the overall performance (net solar-to-electricity and energy density) of the thermochemical storage system on an industrial scale. Different process flow diagrams (PFDs) were compared, involving low (CaO storage at 50 °C) and high (800 °C) temperatures. The analysis is constructed upon the multicycle CaO conversion data reported in the previous section (Table 3). The multicycle CaO conversion was assumed to remain constant, as in cycle 20. Considering the operation of the plant, if the conversion eventually dropped below that level, it could be compensated by introducing a fraction of fresh material (makeup). Furthermore, it was assumed that there is no significant change in multicycle CaO conversion by increasing or decreasing the storage time, as indicated in Section 3.2.

**Figure 8:**
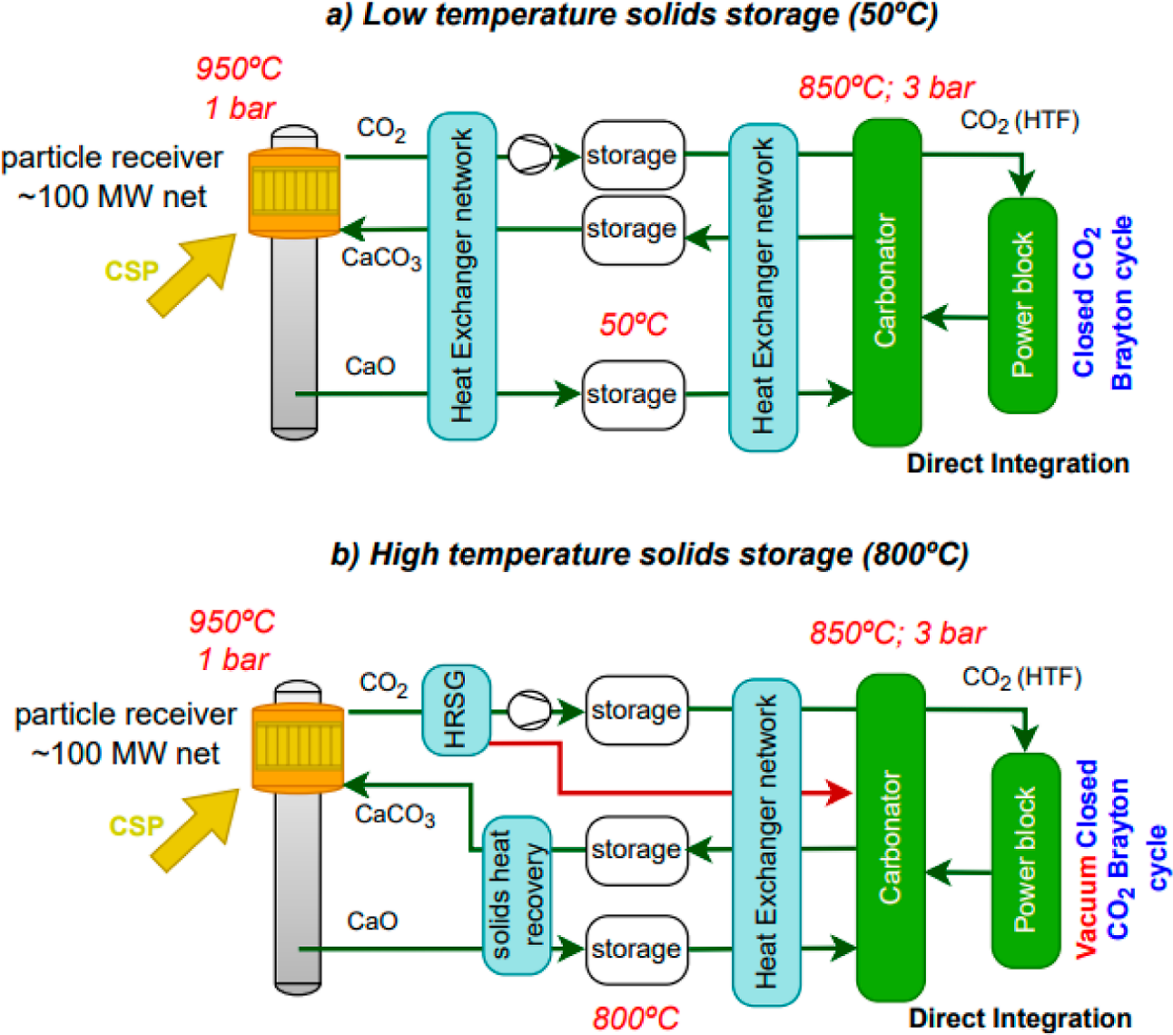
PFDs evaluated: (a) storage at low temperature (based on ref (13)) and (b) storage at high temperature (based on ref (15)).

The temperature in the solid storage vessels constrains the configuration of the process scheme and the efficiency since a more complex heat exchanger network is required in cases with low-temperature solid storage. Table 5 resumes the main assumptions for the CSP CaL schemes at low-temperature and high-temperature storage, which are taken from refs (13) and (15). Figure 8 shows a conceptual representation of these process schemes. Complete information about assumptions and process modeling can be found in the referenced papers since, for the comparison, the original configuration of the PFD is faithfully followed. Low-temperature storage allows energy storage without thermal losses (even in seasonal energy storage). Because of the high temperatures in both the calciner and the carbonator reactors, low-temperature storage involves a significant drop in the temperature of the materials throughout the cycle. It requires an optimized heat integration to achieve adequate system efficiency. When high-temperature solid storage is considered, the process scheme is simplified by requiring fewer heat exchangers because of a lower temperature difference between the reactors and the storage. However, thermal losses increase at high temperatures, as well as problems related to the material’s high-temperature cohesion.^58^ This is not a minor matter as the increase in cohesiveness in the material as the temperature increases^59^ would negatively affect the fluidization of the material to extract it from the storage tank to complete the carbonation cycle. To improve this situation, some coatings, such as silica or titania, could be added to the particles to improve their flowability.^60^ From a life cycle and environmental assessment perspective, there are no significant differences between the storage of solids at high or low temperatures.^43^

**Table 5:** Main Assumptions in the CSP CaL Model

group	parameter	component	value
turbomachinery	isentropic efficiency	compressors, turbines	0.89
	number of intercooling/reheating stages	high pressure storage compressor (HPS-COMP)	5
		main CO_2_ compressor (M-COMP)	3
		CO_2_ turbine (HPS-TURB)	3
	intercooling/reheating temperature	high pressure storage compressor (HPS-COMP)	40 °C
		high pressure storage turbine (HPS-TURB)	65 °C/100 °C
heat exchangers	minimum temperature difference	gas–gas HX	15 °C
		solid–gas HX	15 °C
		solid–solid HX	20 °C
		CO_2_ cooler	20 °C
	pressure drops	coolers	1%
		HXG (both sides)	5%
		HRSG (hot side)	3%
		HRSG (cold side)	11%
		solid–gas HX (both sides)	3%
reactors	efficiency	calciner	1
	heat input	calciner	100 MW
	heat losses	carbonator	1% of heat transferred
storage vessels	temperature losses	all	0 °C
	CO_2_ storage conditions	CO_2_ vessel	75 bar
			25 °C
steam cycle	isentropic efficiency	steam turbine (ST)	0.75
	mechanical–electric efficiencies	steam turbine (ST)	0.98
	condensing pressure	COND	0.075 bar
	evaporation pressure	HRSG	45 bar
	superheated steam temperature	HRSG	400 °C
heat rejection	auxiliaries electric power consumption	all coolers	0.8% of heat released

Given the advantages and disadvantages of storing solids at high temperatures, the cycle performance analysis provides valuable information for the overall process design.

Both PFDs were designed and simulated under steady-state conditions. The different PFDs (Figure 8) were modeled from the process scheme and the assumptions indicated in each reference work. Solar side losses were not considered for comparison since all the schemes are based on the same particle receiver size and temperature. Thus, the net thermal-to-electric efficiency was compared for each case. As is typical in many previous studies,^2^ this efficiency was calculated as a weighted average throughout the day, assuming 8 h of constant solar irradiation in the “sun mode” and 16 h without radiation in the “night mode”. This involves a solar multiple (SM) of 3, with the SM being the receiver design thermal output ratio to the power block design thermal input. Under this simplified approach, the efficiency of the plant was calculated according to eq 8.^19^
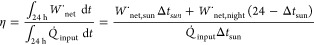
8

where *Ẇ*_net,sun_ and *Ẇ*_net,night_ are the net power produced in “sun” and “night” modes, respectively, Δ*t*_sun_ = 18 h, and *Q̇*_input_ is the net solar power entering the power plant.

Equation 9 has been proposed to describe the overall energy storage capacity of the system to provide a more realistic measure of the energy density of the overall storage system.^15^ It is closely related to plant expenses and is a critical factor for accounting for the size of the vessels needed for both gas and solid storage. Reactors or heat exchangers are not included in the volumetric energy storage density since it is considered only the energy storage stage. Sensible heat stored accounts for around 40% of the high-temperature storage scheme (Figure 8b).
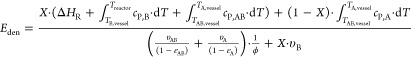
9where *X* is the conversion, Δ*H*_R_ is the reaction enthalpy (GJ/kmol), *c*_*p*,*i*_ is the specific heat of component *i* (MJ/kmol·K), *T*_reactor_ is the decomposition reaction temperature (K), *T*_*i*,vessel_ is the storage temperature of component *I* (*K*), υ_*i*_ is the specific volume of component *i* at storage conditions (m^3^/kmol), ε_*i*_ is the internal porosity of component *i*, and ϕ is the particle packing density, whose value is set to 0.6 as a standard value for the random loose packing fraction of irregularly shaped particles under gravity.

**Figure 9:**
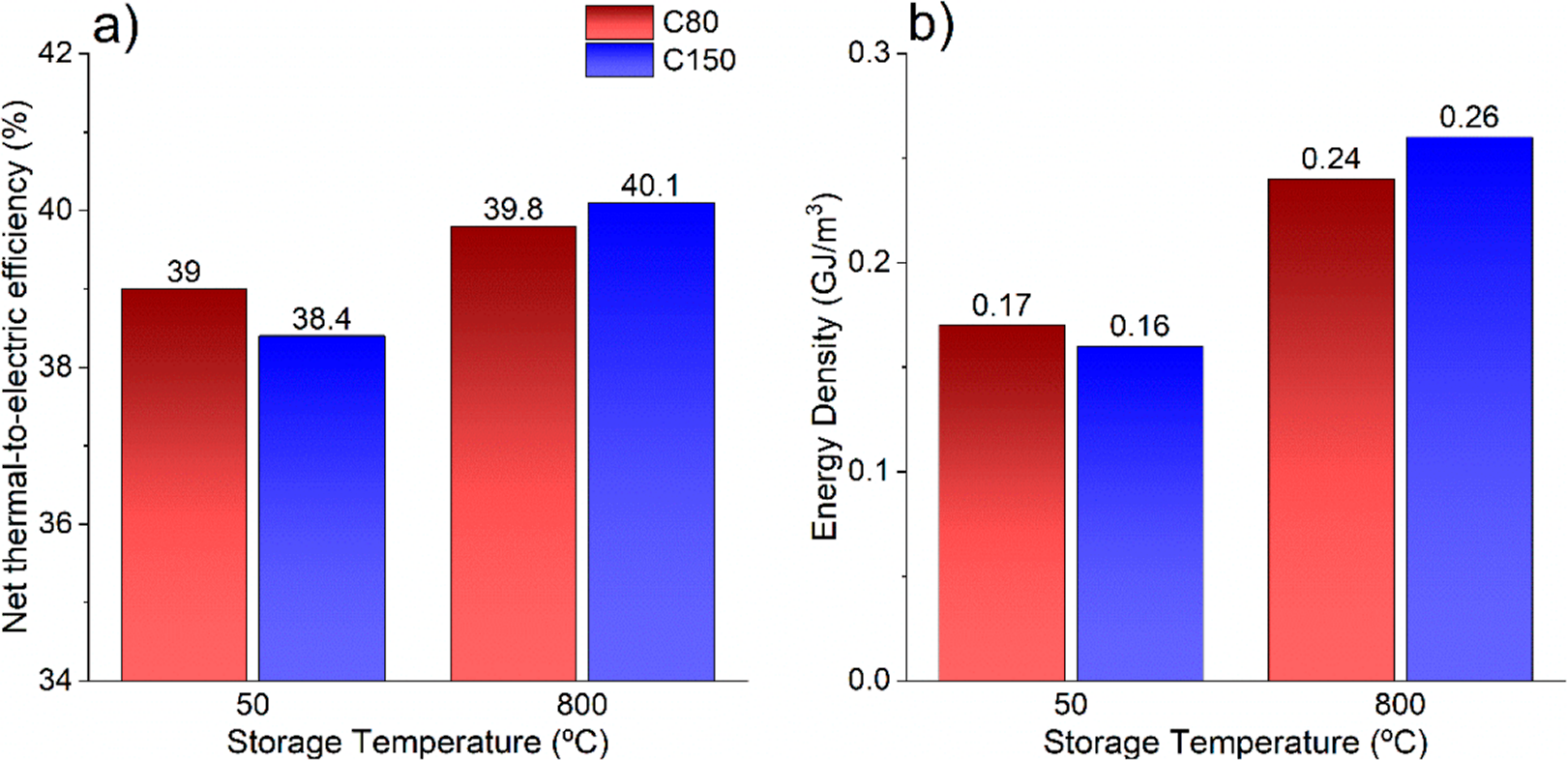
(a) Plant efficiency and (b) overall energy density as a function of the storage temperature and the particle size.

The experimental results (Table 3) show that multicycle CaO conversion does not vary significantly within the 80–150 μm particle size. Regarding temperature, CaO conversion slightly increases when considering low-temperature (and prolonged) solids storage. Figure 9a illustrates the net thermal-to-electric efficiency for the two particle sizes of solids and storage temperatures. By a comparison of the effect of the temperature on the thermal-to-electric efficiency, it can be seen that higher efficiency is achieved at higher storage temperatures. High-temperature storage implies a more straightforward and efficient energy integration process. In any case, the difference in the overall performance for each analyzed case is slight (+2–4%), reinforcing the finding that the system suffers a smooth penalty when storing the material at low temperature (allowing the energy storage in the long term) if adequate energy integration is carried out.

In addition to storing energy in the long term with a reduced energy penalty, storing solids at low temperatures presents a fundamental advantage regarding the flowability of the material. According to ref (58), when increasing the material storage temperature from 25 to 500 °C at a consolidation stress of 1500 Pa, the tensile strength increases from around 50 Pa to above 700 Pa, directly impacting the material flowability. Hourly term storage temperatures higher than 500 °C would involve reaching the behavior of a very cohesive and nonflowing solid,^59^ highly penalizing the process operation.

Regarding the overall energy density (Figure 9b) estimated by eq 9, the value is highly enhanced for high temperature due to the increase in sensible energy storage. Since the CaO conversions for high- and low-temperature storage are similar, there is not much impact associated with the number of solids to be stored (which penalizes the overall energy density). It is important to note that for calculating the required solid storage volume, the packing factor has been considered independent of the temperature (a constant value of 0.6^61^). However, the lab-scale test showed that the packing density smoothly increases with the temperature.^59^

## Conclusions

This work assesses the influence of the implementation of a storage phase on the multicycle performance of CaCO_3_ with different PSDs. The innovation of the study resides in the consecution of the main objective: evaluate the influence of temperature, atmosphere, time, and particle size during the storage step. This allows for the assessment of the long-term energy storage performance, one of the key advantages of thermochemical energy storage systems.

Limestone particles of particle sizes in the range of 80–150 μm were tested for multicycle performance using schemes implementing a storage phase. Three different storage conditions were tested: 200 °C matching molten salts, 50 °C as room temperature in a CO_2_ atmosphere, and 800 °C for industrial plant integration in N_2_. Results show that storage temperatures below 200 °C in CO_2_ result in very slight residual carbonation and are mostly limited to the first few cycles. Thereafter, the decay in reactivity due to the progressive sintering of the material protects the cycled CaO to react significantly during the storage phase. Storage time does not significantly impact the residual carbonation as it mostly occurs the first 5–10 min after CO_2_ injection. For longer periods, the loss of active material becomes negligible. Thus, long-term storage at low temperatures appears to be viable even in a reactive atmosphere such as CO_2_.

For effective CaO conversion, the best performance was obtained for the C80 sample at a low-temperature storage step (at 50 °C, *X*_CaO,20_ = 0.126). However, the difference with the other conditions tested are slight, thereby leading to the conclusion that there is limited dependence of conversion on the storage step.

From a process engineering perspective, storing solids at low temperatures can significantly improve their flowability, which could have a crucial effect on the overall process operation. Although a higher storage temperature facilitates energy integration and system efficiency, the analysis results show that the effect of storing solids at ambient temperature is only 2–4% less as compared with that at high temperature. These results confirm the potential of the CaL process as a storage system in the long term. The lower energy density of the system when storing at low temperatures (losing the contribution of storing sensible heat) could be compensated by introducing a certain fraction of limestone makeup to enhance the average multicyclic conversion, which would considerably improve the energy storage density.

The results obtained are of utmost importance because the CaL system’s main advantage is the possibility of storing the products of the *reaction* in the long term. Understanding this stage is fundamental for a profound knowledge of the CaL TCES system. Moreover, the results presented in this work could help in the development of more realistic engineering models.
